# A low-cost DAC BIST structure using a resistor loop

**DOI:** 10.1371/journal.pone.0172331

**Published:** 2017-02-17

**Authors:** Jaewon Jang, Heetae Kim, Sungho Kang

**Affiliations:** Dept. of Electrical and Electronic Engineering, Yonsei University, 50 Yonsei-ro, Seodaemoon-Gu, Shinchon-Dong 134, Seoul, Korea; Nanjing Normal University, CHINA

## Abstract

This paper proposes a new DAC BIST (digital-to-analog converter built-in self-test) structure using a resistor loop known as a DDEM ADC (deterministic dynamic element matching analog-to-digital converter). Methods for both switch reduction and switch effect reduction are proposed for solving problems related to area overhead and accuracy of the conventional DAC BIST. The proposed BIST modifies the length of each resistor in the resistor loop via a merging operation and reduces the number of switches and resistors. In addition, the effect of switches is mitigated using the proposed switch effect reduction method. The accuracy of the proposed BIST is demonstrated by the reduction in the switch effect. The experimental results show that the proposed BIST reduces resource usages and the mismatch error caused by the switches.

## Introduction

With the development of new applications, the performance of DACs (digital-to-analog converters) and ADCs (analog-to-digital converters) has become increasingly important [1]. High resolution DACs and ADCs which have small voltage range are recently used for analog circuits, and it decreases the minimum unit of voltage [2, 3]. The analog circuits with the small unit of voltage is more influenced by errors such as voltage drops or glitches. DAC testing is quite complex because additional test circuitry with high accuracy is required [4]. To test high-performance DACs, achieving the same resolution as that obtained with the ATE (automatic test equipment) must be possible. However, such an ATE is not always manufactured on a commercial scale because of cost concerns. To solve this problem, BIST approaches have been proposed. BIST structures not only improve the efficiency and the application time of tests but also eliminate the use of expensive external ATE. BIST architectures are characterized by a complicated structure and high hardware overhead requirements due to the need for additional circuitry. A DAC BIST architecture with PWM (pulse-width modulation) and two sinusoidal carriers was proposed in [5]. However, the structure requires high area overhead and complex circuits for implementation.

To address the above problems, DEM (dynamic element matching) methods were researched while those using multiple elements (DACs, ADCs, modulators or resistors), and coarse elements are used for decreasing hardware overhead. The coarse elements decrease the accuracy of the circuit, so they produce the same outputs with various ways for averaging out errors. In [6], the DEM method is used for the implementation of high SFDR (spurious-free dynamic range) multibit DSMs (delta–sigma modulators). However, this method causes high hardware overhead and cannot be applied to ADCs or DACs. For testing a high-speed DAC, a DEM ADC uses multiple low-cost ADCs or resistors rather than a single high-performance high-cost ADC. Due to process variations, mismatch errors inevitably arise in integrated circuits. However, the DDEM (deterministic dynamic element matching) technique arranges matching-sensitive elements to generate appropriate outputs. In [7], a DDEM method with dithering was investigated for high-performance DAC testing with low-resolution flash ADCs. The method uses resistor string that 2^n^ resistors and 2^*n*^ + 1 switches in series. Such an approach suffers from mismatch errors by the number of switches. In [8], a DEM flash ADC architecture that had no switches between resistors was examined. However, the method still produces mismatch errors because switches between voltage source and resistors still produce the errors. [7, 8] used low-cost DDEM structures to test ADCs or DACs. While the DDEM ADC structure has been improved over many years, it still has hardware overhead problems and mismatch errors due to switch effects. [9] proposes a method that uses less resistors and switches by adopting a switch reduction method. While the method can reduce hardware overhead, mismatch errors that decrease the accuracy of DDEM ADC still exist.

In this paper, a new algorithm that reduces the number of switches and switch effects is proposed. The suggested architecture makes DDEM ADCs more accurate and solves the hardware overhead problem.

## Conventional DDEM ADC method

**Fig 1:**
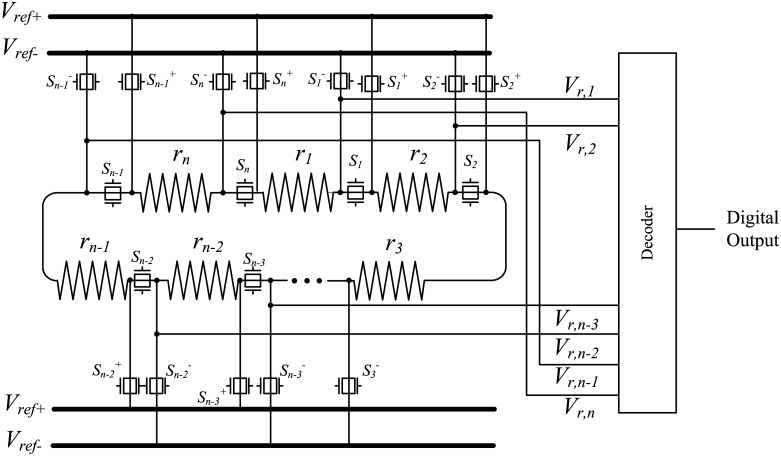
Structure of a conventional *k*-bit DDEM ADC.

In this work, a DDEM ADC architecture that includes a resistor loop is proposed. An example of the structure of a conventional k-bit DDEM ADC (*k* = ⌈log_2_ *n*⌉) is shown in Fig 1. The DDEM ADC produces different reference voltages at every interconnection between adjacent resistors [10]. The rearrangement of resistors in the resistor loop allows the generation of different outputs. The resistors are physically connected as a loop via switches and the loop can be broken at different positions by opening specific switches to build different resistor strings. The generation of reference voltages is possible through the transitions of the switch control signals. There can be *P* types of switch control signals, where *P* is the number of different resistor strings and the divisor is 2^k^.

A set of reference voltages is generated by connecting the two nodes of the switches (*S_i_*^+^, *S_i_*^−^) to external reference voltages. Therefore, *P* digital outputs are available for one analog input, and the average of *P* digital outputs is the quantized output of the DDEM ADC. For example, when *S*_1_ is opened, switches from *S*_2_ to *S*_*n*−1_ and *S*_*n*_, as well as switches *S_i_*^+^ and *S_i_*^−^ must be closed and other switches must be opened. Reference voltages *V*_1_, *V*_2_ to *V*_*n*_ then become the reference voltages in the descending order.

We used a DDEM ADC to test DAC nonlinearities. A *k*-bit DDEM ADC needs at least *n* unit resistors with resistances that are all 1 for a resistor string. Each comparator yields a value of “1” when the analog input voltage is higher than the reference voltage; otherwise, the output of the comparator is “0.” The decoder shown in Fig 1 converts binary codes from comparators to an appropriate digital output.

As a conventional DDEM ADC always produces the same voltages, the loop resistor form is used [11]. To convert a resistor loop into a resistor string, one switch (e.g., *S*_1_) is opened and the two ends are connected to positive and negative references. Different *P* switches should be selected as the break points in the loop so as to yield *P* different configurations. In the DDEM algorithm, these selected P switches are treated as two end points and are connected to reference voltages (*V*_*ref*+_, *V*_*ref*−_). For example, if *P* is equal to 4, the first configuration is generated by opening *S*_1_, as shown in Fig 1, and the second, third, and fourth configurations can be generated by opening *S*_2_, *S*_3_, and *S*_4_, respectively. Therefore, for each digital output code, the DDEM ADC generates four corresponding outputs. The four transition points for input code 5 will be as follows.

$$\begin{array}{c} T_{5}^{1}=\frac{\left(V_{ref+}-V_{ref-}\right)}{\sum_{k=1}^{2^{n}}R_{k}}\left(r_{1}+r_{2}+r_{3}+r_{4}+r_{5}\right)\\ T_{5}^{2}=\frac{\left(V_{ref+}-V_{ref-}\right)}{\sum_{k=1}^{2^{n}}R_{k}}\left(r_{2}+r_{3}+r_{4}+r_{5}+r_{6}\right)\\ T_{5}^{3}=\frac{\left(V_{ref+}-V_{ref-}\right)}{\sum_{k=1}^{2^{n}}R_{k}}\left(r_{3}+r_{4}+r_{5}+r_{6}+r_{7}\right)\\ T_{5}^{4}=\frac{\left(V_{ref+}-V_{ref-}\right)}{\sum_{k=1}^{2^{n}}R_{k}}\left(r_{4}+r_{5}+r_{6}+r_{7}+r_{8}\right) \end{array}$$ (1)

where *R*_*k*_ is the resistance of the *k*-th resistor in the resistor loop.

## Proposed BIST

In conventional DDEM ADCs, all resistors have the same resistances. Therefore, a *k*-bit ADC requires 2^k^ resistors and 3 × 2^*k*^ switches. Consequently, *n*(= 2^*k*^) types of reference voltages can be obtained. The number of switches increases as the resolution of the DDEM ADC increases. Such an increase causes an area overhead problem. Switch reduction methods, which modify the resistance of the resistors in the resistor loop, have been proposed to solve this problem. However, these solutions may reduce the accuracy of the DDEM ADC. Therefore, the switch effect reduction method will be performed after the switch reduction method so as to improve the accuracy of the DDEM ADC. The two methods require different *P*s.

### Switch reduction technique

**Fig 2:**
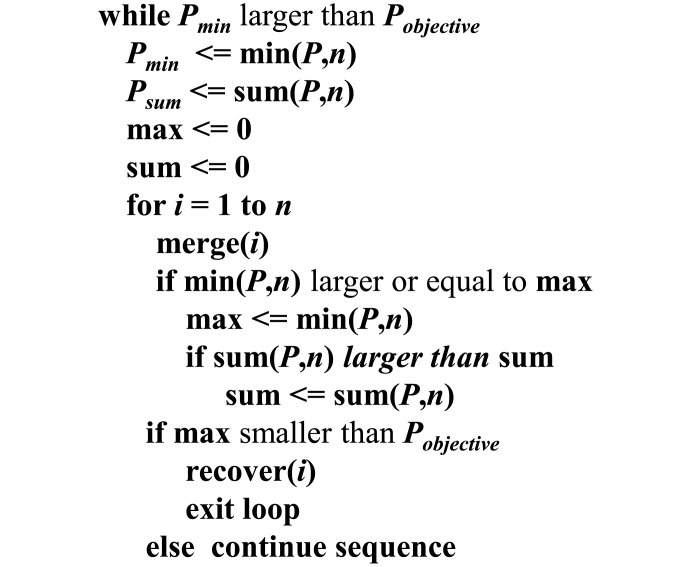
Pseudocode of the switch reduction method.

One of the main goals of this work is to reduce the hardware overhead in a resistor string. Each switch is connected between a resistor and a reference voltage (*V*_*ref*+_ or *V*_*ref*−_) and the resistance of every resistor is equal to 1. The number of switches depends on the number of resistors in the DDEM ADC. Thus, if the number of resistors is reduced, the number of switches is also reduced. Output voltages are produced in the ratio of the resistance of all resistors to the resistance of the resistors from *V*_*ref*+_ to the output node. Therefore, if the resistances of some resistors are increased, the number of resistors is reduced because the sum of all resistors is fixed at 2^k^. The pseudocode for the proposed BIST is shown in Fig 2. The scheme reduces the number of resistors in the resistor string by maintaining a high value of *P*. To be effective, the algorithm must have an objective *P*_*min*_ value (*P*_*objective*_). The proposed BIST reduces the number of resistors until the objectives are reached. The resistances of merged resistors are increased to reduce the number of switches and maintain the total resistance. However, if the resistance of some resistors is increased, the value of *P* will decrease. The value of *P* must be maintained at a certain level because *P* affects the accuracy of the DDEM ADC.

**Fig 3:**
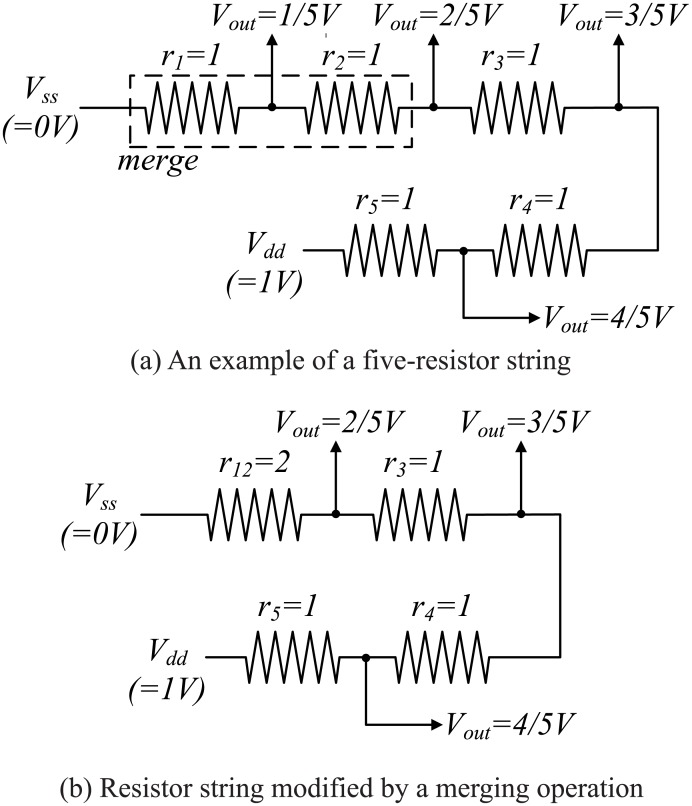
An example of a merging operation.

The resistance of each resistor is initially 1, and there are edges between adjacent resistors. If one edge is selected, the two resistors that are adjacent to the edge are merged into one resistor with a resistance that is the same as the sum of the two resistors. As a result, *P* is reduced. An example of this phenomenon is shown in Fig 3. Assume that there are only five resistors and that the resistance of each resistor is equal to 1. Next, select one edge that connects two adjacent resistors. If the edge is broken, the resistor loop transforms into the resistor string shown in Fig 3(a). This resistor string can produce four different output voltages: 1/5, 2/5, 3/5, and 4/5. If two resistors are merged, the resistance of the merged resistor will be the sum of the resistances of the two resistors. If one edge is selected as described above, the resistor loop is changed to that shown in Fig 3(b). This resistor string can only produce three different output voltages: 2/5, 3/5, and 4/5. Such a strategy not only reduces the number of different outputs but also decreases the value of *P*. The example shown in Fig 3 indicates that the merging operation decreases both the value of *P* and the accuracy of the DDEM ADC.

The costs of the edges are defined by *P*_*min*_. The following procedures are used to calculate *P*_*min*_. First, the edge between the first resistor and the second resistor is selected. It is assumed that the edge is broken, that the first resistor is connected to *V*_*ref*+_, and that the second resistor is connected to *V*_*ref*−_. Next, the resistances of sets of resistors that are adjacent to each other and include the first resistor are added. The above two processes are then repeated until all edges are selected and the value of *P*_*min*_ is calculated.

**Fig 4:**
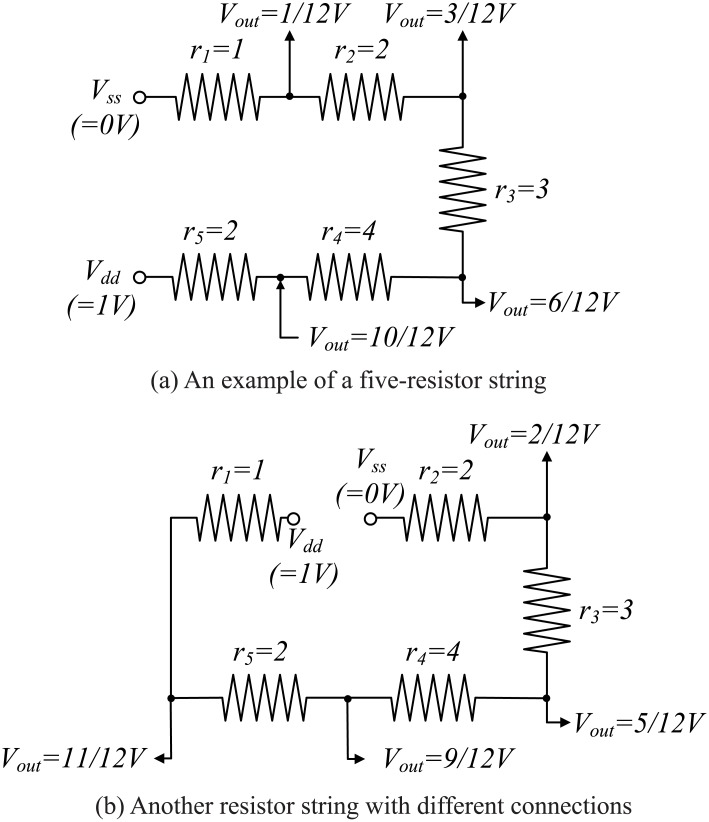
Resistor string change with different connections.

The same resistor string with different connections between resistors and the reference voltages is shown in Fig 4. The resistances of *r*_1_, *r*_2_, *r*_3_, *r*_4_, and *r*_5_ in the figure are 1, 2, 3, 4, and 2, respectively. The first resistor (*r*_1_) is connected to *V*_*ref*+_ while the last resistor (*r*_5_) is connected to *V*_*ref*−_; the total resistance of all resistors is 12. The initial resistor string is shown in Fig 4(a). This resistor string can produce four different output voltages: 1/12, 3/12, 6/12, and 10/12. If the second resistor (*r*_2_) is connected into *V*_*ref*+_ and the first resistor is connected to *V*_*ref*−_, the resistor string is changed to that shown in Fig 4(b). This resistor string can produce four different output voltages: 2/12, 5/12, 9/12, and 11/12. In this manner, different output voltages for all edges can be found and the number of different types for generating the same output voltage becomes *P*.

### Switch effect calculation

A DDEM ADC with a resistor loop produces output voltages by closing and opening switches. In every clock, the resistor loop becomes a resistor string where resistors and switches are connected between reference voltages.

**Fig 5:**
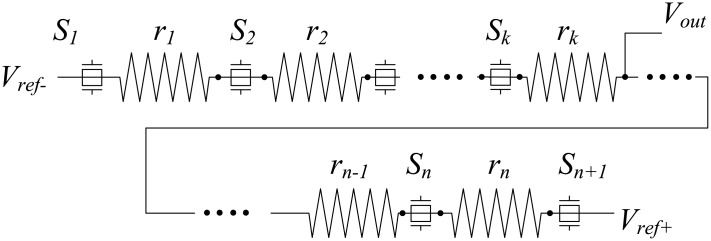
An example of a resistor string form.

An example of a resistor string form with n resistors is shown in Fig 5. The ideal output voltage *V*_*ideal*_ is given as follows.

$$V_{ideal}=\left(V_{ref+}-V_{ref-}\right)\times \frac{\sum_{m=1}^{k}r_{m}}{\sum_{m=1}^{n}r_{m}}$$

However, there are *n* resistors and *n* + 1 switches between the reference voltages while there are *k* resistors and *k* switches between *V*_*ref*−_ and *V*_*out*_ node. The switches can cause voltage drops. The measured output voltage, *V*_*out*_, is expressed as follows.

$$V_{out}=\left(V_{ref+}-V_{ref-}\right)\times \frac{\sum_{m=1}^{k}r_{m}+\sum_{m=1}^{k}sw_{m}}{\sum_{m=1}^{n}r_{m}+\sum_{m=1}^{n+1}sw_{m}}$$

where *sw*_*i*_ denotes the resistances of switch *i* when switch causes a voltage drop. It is assumed that switch effect error (*SE*) is the difference between ideal output voltage (*V*_*ideal*_) and measured output voltage (*V*_*out*_) as follows.

$$SE=\frac{\sum_{m=1}^{k}sw_{m}}{\sum_{m=1}^{n+1}sw_{m}}-\frac{\sum_{m=1}^{k}r_{m}}{\sum_{m=1}^{n}r_{m}}$$

The switch effect error may be defined as the error caused only by mismatches in the number of switches. The switch effect error influences the difference between the ideal output voltage and the measured output voltage. The more the switch effect is reduced, the closer the measured output voltage is to the ideal output voltage.

A conventional DDEM ADC has unit resistors and thus the resistance of each resistor is 1 and the number of resistors is not reduced. If we assume that the normalized switch effect in previous work is *SE*_*prev*_, then the normalized switch effect in input code *i* can be calculated as follows.

$$SE_{prev}\left(i\right)=\frac{i}{n}-\frac{i}{n+1}=\frac{i}{n\cdot (n+1)}$$

**Fig 6:**
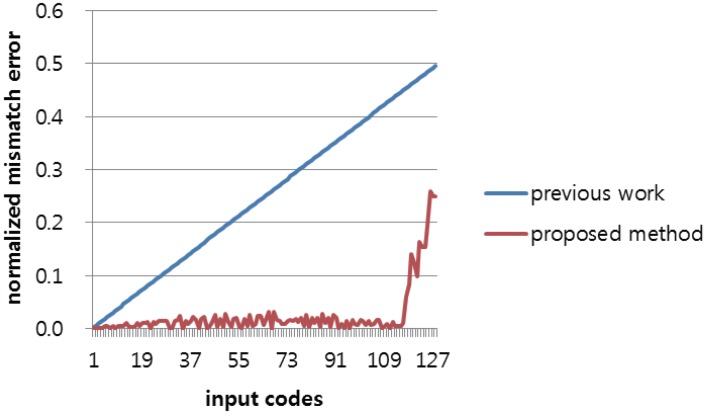
A mismatch error comparison graph.

The mismatch error will increase due to an increase in input code *i*. If *i* were *n*, then the error would reach its maximum value. However, the error decreased in every output code when the proposed BIST was applied. An error comparison graph between the proposed BIST and previous work [8] for a 7-bit DDEM ADC is shown in Fig 6.

The error in the switch effect is not related to the value of *P* and hence the errors cannot be averaged out. This error is always the same regardless of the resolution of the DAC. The switch reduction method can force various numbers of switches to have the same resistance. The errors can then be averaged out if the number of switches is varied in one output code. This switch effect error influences the accuracy of the DDEM ADC. Reducing the switch effect is also important because a portion of the switch effect error will be increased when smaller or poorer switches are used for the resistor loop.

### Switch effect reduction method

**Fig 7:**
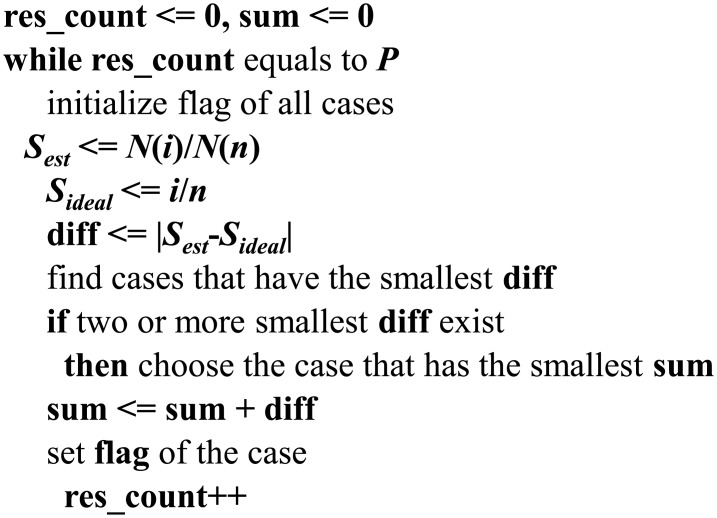
Pseudocode of the switch effect reduction method.

Another primary goal of this work is to determine a strategy to reduce the effects of the switches. There are resistors with various resistances from *V*_*ref*−_ to the output code, but the number of switches is not used for averaging. Therefore, the cases that can reduce the switch effect are selected by the proposed switch effect reduction method. The pseudocode for the method is shown in Fig 7. This pseudocode, which is performed after the switch reduction method, calculates the number of switches between *V*_*ref*−_ and the input code *i*, *N*(*i*), and determines the case that has the smallest normalized switch effect error.

For example, a 6-bit DDEM ADC has 64 resistors and thus there are 65 switches from *V*_*ref*+_ to *V*_*ref*−_. We assume that the number of switches can be reduced from 65 to 50 by the switch reduction method, and the numbers of switches for output code 49 are as follows:

SW={39,38,39,39,40,38,…,38,39,39,40,39}

In the case of output code 49, the ideal average number of switches from *V*_*ref*−_ to the output node is 49 when the number of switches is 64 and the value of *P* is 32. Therefore, the ideal number of switches between *V*_*ref*−_ to the output node is 24.5.

$$N{\left(49\right)}_{ideal}=\frac{49}{64}\times 32=24.5$$

However, the number of switches is 65 and the number of switches from *V*_*ref*−_ to the output node is 49. The switch effect will be 24.123 for conventional DDEM ADCs. Therefore, the mismatch error will be 0.877.

$$N{\left(49\right)}_{prev}=\frac{49}{65}\times 32=24.123$$

In the proposed switch effect reduction method, the following 32 cases of switches are selected.

$$SW_{selected}=\left\{39,39,39,39,39,39,39,39,38,38,...,38\right\}$$

$$N{\left(49\right)}_{prop}=\frac{38\times 24+39\times 8}{65}=24.48$$

The value obtained with the proposed switch effect reduction is closer to the ideal value than that generated in previous work. Such a result is evidence of the superior performance of the proposed switch effect reduction method in reducing the switch effect.

## Results

The proposed DDEM ADC structure is verified by C/C++ and HSPICE simulations. In the simulation, DDEM ADCs from 6-bit to 12-bit are modeled as devices under testing, and the *P*_*objective*_ is set to 2^*k*−1^ for *k*-bit DDEM ADC. The reduction in the switch effect and the numbers of switches and resistors are calculated. The resistor strings are generated by resistors from a Gaussian distribution with a nominal value of 1 and *σ* = 0.1. In addition, the simulation uses 0.35*μm* MagnaChip library [12], and the current-steering DACs which use current sources are used as the benchmark circuit [13]

A comparison of the area overheads derived in this work and those presented in the previous researches [8] and [9] is shown in Table 1. The previous study examined conventional flash DDEM ADCs with a resistor loop; the resistances of all resistors were equal to 1. Because the *n*-bit DDEM ADC requires 2^n^ resistors and 3 × 2^*n*^ switches, and each switch occupies 4.4*μm*^2^ in MagnaChip library. The previous study used the same number of resistors and switches regardless of *P*. For example, a 6-bit DDEM ADC requires 844.8*μm*^2^ and 64 resistors, while a 7-bit DDEM ADC requires 1689.6*μm*^2^ and 128 resistors. However, the DDEM ADC developed with the proposed BIST requires less area overhead in every case. Therefore, the proposed BIST reduces the required number of area overhead by about 16.35% compared to the conventional method. In addition, the proposed BIST has similar area overhead to that in previous work [9]

**Table 1:** Hardware overhead comparison with previous works (*μm*^2^).

Resolution	Conventional [8]	Previous work [9]	Proposed BIST
6-bit	844.8+64 resistors	646.8+64 resistors	646.8+49 resistors
7-bit	1689.6+128 resistors	1412.4+128 resistors	1412.4+107 resistors
8-bit	3379.2+256 resistors	2824.8+256 resistors	2785.2+211 resistors
9-bit	6758.4+512 resistors	6006.0+455 resistors	5583.6+423 resistors
10-bit	13516.8+1024 resistors	12342.0+935 resistors	12236.4+927 resistors
11-bit	27033.6+2048 resistors	23258.4+1762 resistors	23245.2+1761 resistors
12-bit	54067.2+4096 resistors	46450.8+3519 resistors	43850.4+3322 resistors

If the numbers of resistors and switches are reduced, the switch effect becomes problematic. The switch effect is derived from the ratio of the number of all switches to the number of switches. To reduce the switch effect, this ratio which is included from *V*_*ref*−_ to the output node must be close to the ratio of the output voltage to the full voltage scale.

The switch effect can be reduced because the DDEM ADC averages *P* types of output voltages. Therefore, the switch effect will be reduced when the average ratio of the switches is close to the ideal ratio (the ratio of the output voltage to the voltage of the full voltage scale).

In the conventional DDEM ADC, the error voltages are fixed to 0.2375 LSB regardless of the P value or the resolution of the DDEM ADC. The results of error voltages obtained with the proposed BIST are summarized in Table 2. The proposed BIST reduces error voltages for every resolution of the DDEM ADC. Therefore, Table 3 shows the error voltages per LSB for calculating the accuracy of the proposed BIST. The previous work [9] uses small area overhead that requires a similar number of resources to that of the proposed BIST, but the switch effects are even higher than the conventional method.

**Table 2:** Error voltage comparison with previous works (*mV*).

Resolution	Conventional [8]	Previous work [9]	Proposed BIST
6-bit	6.12305	1.64324	0.69408
7-bit	3.06152	3.37560	0.55907
8-bit	1.53076	1.39058	0.09145
9-bit	0.76538	2.04678	0.16756
10-bit	0.38269	1.57460	0.04666
11-bit	0.19135	0.23877	0.01657
12-bit	0.09567	0.22578	0.01675

**Table 3:** Error voltage comparison with previous works (LSB).

Resolution	1 LSB(*mV*)	Conventional [8]	Previous work [9]	Proposed BIST
6-bit	25.78125	0.23750	0.06374	0.02692
7-bit	12.89063	0.23750	0.26187	0.04337
8-bit	6.44531	0.23750	0.21575	0.01419
9-bit	3.22265	0.23750	0.63512	0.05199
10-bit	1.61132	0.23750	0.97721	0.02896
11-bit	0.80566	0.23750	0.29636	0.02057
12-bit	0.40283	0.23750	0.56048	0.04159

**Fig 8:**
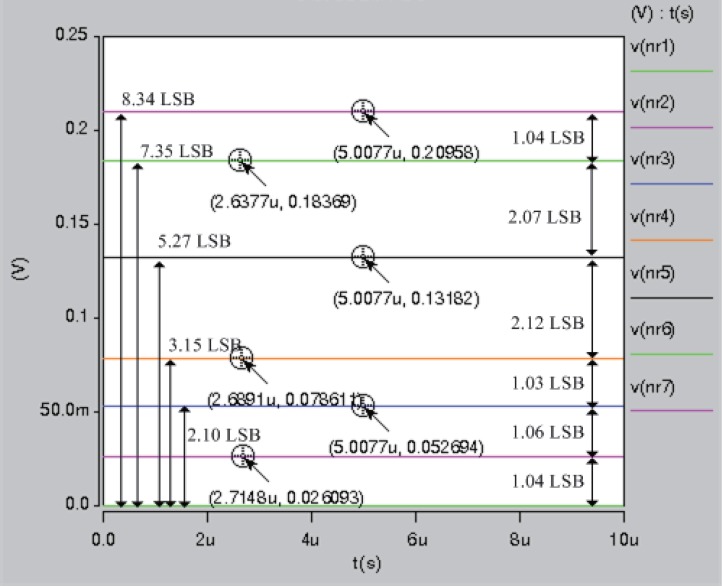
Spice simulation result in 7-bit DDEM ADC with *P* = 64.

In HSPICE simulations, we implemented a 7-bit DDEM ADC. The value of *P* was equal to 64 and 1 LSB (Least Significant Bit) was 12.89 *mV* as shown in Fig 8. The first six resistors in the resistor string exhibited resistances of *r*_1_ = 1, *r*_2_ = 1, *r*_3_ = 1, *r*_4_ = 2, *r*_5_ = 2, and *r*_6_ = 1. The differences in the outputs were 1 LSB, 1 LSB, 1 LSB, 2 LSB, 2 LSB, and 1 LSB with small mismatch errors (less than 0.2 LSB). The results indicate that switch errors are canceled using the proposed BIST. The mismatch errors ranged from -0.2812 LSB to 0.3812 LSB in the 7-bit DDEM ADCs. The maximum error due to the switch effect and resistor mismatch is limited to 1/2 LSB or -1/2 LSB. Thus, the accuracy of the proposed DDEM ADC structure is guaranteed.

## Discussion

In this paper, a DDEM ADC structure suitable for testing high-performance DACs was described. The switch reduction method proposed here allows the hardware overhead problems of DDEM ADC structures to be overcome. With the switch effect reduction method, the error caused by switches is reduced. The experimental results indicate that the proposed BIST is feasible for testing high-performance DACs with low hardware overhead. In addition, it was shown that the proposed structure can be applied to non-ideal circuits. Such characteristics make this work a practical solution for realizing a high-resolution DAC BIST structure.

## Supporting information

S1 File Switch effect comparison for all voltage sources. The data contain switch effect from 6-bit DDEM ADC to 12-bit DDEM ADC in the conventional method, previous work and proposed BIST. Every sheet shows the switch effect for all voltage sources (1/2^*n*^ to 2^*n*^/2^*n*^) while *n* is the resolution of the DDEM ADCs.
(XLSX)
